# Exogenous Melatonin Alleviated Leaf Yellowing via Inhibiting Respiration and Ethylene Biosynthesis during Shelf Life in Pakchoi

**DOI:** 10.3390/plants11162102

**Published:** 2022-08-12

**Authors:** Nan Wang, Huixin Fang, Qingxi Yang, Zhiyong Liu, Hui Feng, Shujuan Ji

**Affiliations:** 1 College of Food Science, Shenyang Agricultural University, Shenyang 110866, China; 2 College of Horticulture, Shenyang Agricultural University, Shenyang 110866, China

**Keywords:** pakchoi, shelf life, melatonin, chlorophyll degradation, respiration, ethylene

## Abstract

Postharvest yellowing of leafy plant is a manifestation of senescence, and melatonin (MT) is known to delay leaf senescence in some higher plants. Herein, we investigated the effect of exogenous MT treatment on postharvest pakchoi by monitoring the ethylene biosynthesis and respiratory metabolism. Results showed that exogenous MT effectively extended the shelf life, delayed leaf yellowing, minimized the alteration in Fv/Fm ratio and maintained higher integrity of chloroplast in pakchoi. There was a significant correlation between yellowing index, respiration rate and ethylene production. MT treatments greatly delayed the yellowing process of pakchoi that was associated with the reduced activity of glycolysis pathway and tricarboxylic acid cycle (TCA), increased proportion of pentose phosphate pathway (PPP) in respiratory metabolism, as manifested by the lower activity of phosphohexose isomerase (PHI), succinate dehydrogenase (SDH) and cytochrome C oxidase (COX), downregulated the expression of their corresponding genes, but enhanced the activity and expression level of 6 phosphogluconate dehydrogenase (6PGDH). MT also markedly maintain chlorophyll content by inhibiting ethylene production and action during shelf life, likely a consequence of reduced activities of 1-aminocyclopropane-1-carboxylate (ACC) synthase (ACS) and ACC oxidase (ACO), as well as the expression levels of their related genes. These results collectively indicate that melatonin alleviated leaf yellowing of postharvest pakchoi might be attributed to the suppression of the ethylene biosynthesis and respiratory metabolism, and our findings contribute to provide a good candidate measure for extending shelf life and reducing postharvest loss of pakchoi.

## 1. Introduction

Pakchoi (*Brassica campestris* L. ssp. *chinensis*), known as a green vegetable and often marketed as bunches of shoots, is an important leafy vegetable in Eastern Asia. As a major popular vegetable, pakchoi is palatable and rich in vitamin and minerals. However, its delicate and open leaf is prone to deterioration during the transportation and shelf life after harvest, manifested as leaf yellowing, rot and wilting [1], which leads to inferior sensory quality and loss of commercial value [2,3]. The yellowing caused by rapid chlorophyll degradation is generally regarded as biological markers of leaf senescence [4]. Therefore, retarding the senescence process might be an effective strategy for maintaining the quality of leafy vegetables.

Respiration is an important metabolism progression during storage for postharvest products since the respiration rate contributes to the postharvest senescence and rapid deterioration [5,6]. The aerobic respiration in plants comprises different pathways, mainly including the Embden-Meyerhof-Parnas (EMP) pathway, the TCA cycle, PPP, and the cytochrome C oxidase (COX) pathway [7,8,9]. PHI, SDH, 6PGDH and COX are the main enzymes among the above respiratory pathways [10]. PHI, as a vital enzyme of the EMP pathway, converts the glucose-6-phosphate into fructose-6-phosphate. 6PGDH catalyzes the dehydrogenation of glucose-6-phosphate in PPP pathway. SDH oxidizes succinate to fumarate and is usually used to evaluate the efficiency of TCA cycle [11]. COX functions in electron transfer and energy generation [12]. Li et al. [13] reported that the altered respiratory activity resulted from the defect of enzymes mentioned above could influence the progression of senescence and the quality in broccoli. There was extensive research into methods to extend the shelf life of *Brassica* species by delaying postharvest respiration. Modified and controlled atmospheres (50% O_2_ + 50% CO_2_) delayed yellowing and extended the storage period of broccoli by reduced the activity of SDH and increased the 6PGDH activity [14]. The low temperature treatment had a significant effect on retarding respiratory activity and chlorophyll loss in broccoli [15]. Recently, the use of hydrogen sulphide (H_2_S) and 1-methylcyclopropene (1-MCP) has been verified effective in inhibiting respiration rate and maintaining green leaves in pakchoi [16]. Tan et al. [6] found that melatonin treatment could decrease tissue weight loss and maintain quality by inhibiting respiration rate of Chinese flowering cabbage.

The initiation and progression of senescence is mediated by various phytohormones [17], among which ethylene has received the most attention. According to the stay-green phenotype of an ethylene-insensitive mutant (*etr1*-*1*) defect in ethylene receptor gene ETR, ethylene was verified to be involved in chlorophyll degradation during leaf senescence [18]. The role of ethylene in inducing postharvest chlorophyll loss has been well documented in many studies: Amir-Shapira et al. [19] found that chlorophyll loss was induced by exogenous ethylene in parsley and exogenous ethylene accelerated the yellowing process in spinach leaves [20]. Similarly, chlorophyllase (chlase) activity was increased in ethylene-treated broccoli [21]. Thus, avoiding exposure to ethylene or trying to minimize ethylene production was the best strategy to extend postharvest shelf life and reduce green color loss for a number of brassicas during harvest, storage and transport [1,22]. In broccoli (*Brassica oleracea* var. *italica* L.), the activity of 1-aminocyclopropane-1-carboxylic acid oxidase (ethylene precursor) was increased prior to yellowing and subsequent ethylene synthesis was concurrent with yellowing [23]. 1-MCP could delay broccoli yellowing by decreasing chlase activity [21]. Gapper et al. [24] found that the postharvest broccoli yellowing process was delayed by inhibiting ethylene biosynthesis with cytokinin treatment. Similar result was reported by Able et al. [23] who showed that 1-MCP suppressed ethylene production and loss of green color. The H2S, as another new gaseous ethylene antagonist, reduces the endogenous ethylene production and delays leaf yellowing of the green leafy vegetable [16].

Melatonin (N-acetyl-5-methoxytryptamine), a low molecular weight indole compound, was discovered in 1958 in the bovine pineal gland [25]. Since then, it has been studied extensively and many advances in functions of melatonin have been reported in plants [26,27]. As a master regulator, it played significant roles, including growth, development and senescence [28,29]. It has been considered as a plant bio stimulator against biotic and abiotic stress. It could also regulate processes of plant vegetative development such as flowering, rooting, photosynthetic physiology and biomass yield, leaf senescence and fruit ripening after postharvest [30,31,32,33]. It was shown that exogenous melatonin could improve the postharvest quality of tomatoes [34] and strawberries [35,36]; postharvest treatment with melatonin could delay peach senescence [37,38] and decrease cassava physiological deterioration during storage [39]. In addition, melatonin has been shown to delay leaf senescence in Chinese flowering cabbages [40,41]. Melatonin increased the bioactive substances while delaying yellowing of broccoli [42]. To our knowledge, there is still no information regarding the effect of melatonin on postharvest leaf senescence in pakchoi, and its underlying regulatory mechanism remains unknown.

To this end, the widely cultivated pakchoi cultivar ‘huaguan’ was selected to investigate the responses of pakchoi to MT. The postharvest leaf color appearance, chlorophyll content and chlorophyll fluorescence changes were monitored; the chloroplast ultrastructure was observed; and respiration rate, ethylene production, the enzyme activity involved in respiratory metabolism and ethylene biosynthesis, as well as their related gene expression level were measured. This work might provide a new method to extend the shelf life of pakchoi and lay a foundation for further exploring the molecular mechanism of melatonin-delayed postharvest senescence of this important leafy vegetable. 

## 2. Results 

### 2.1. Exogenous Melatonin Alleviated Leaf Yellowing of Pakchoi

**Figure 1:**
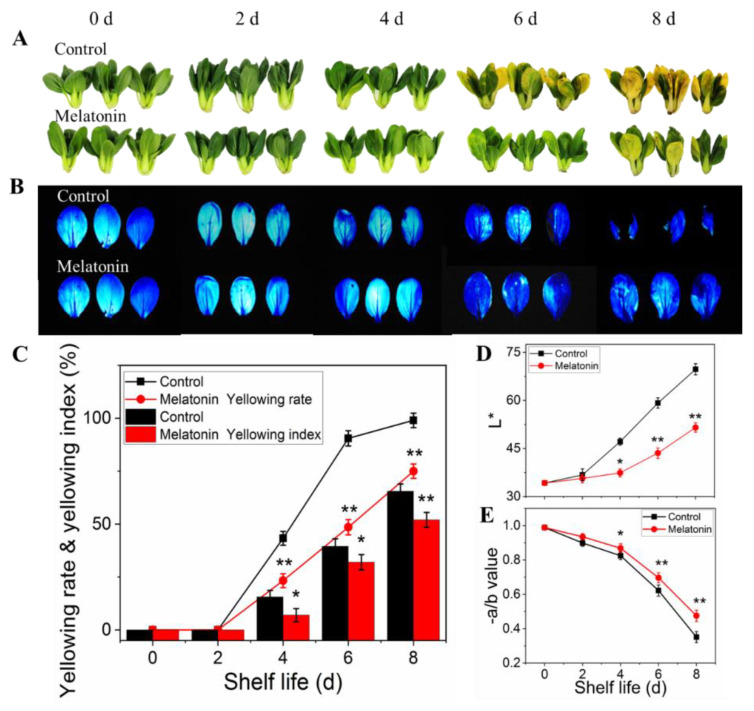
Effects of exogenous melatonin on leaf color changes in postharvest pakchoi. The leaf color phenotype (**A**), chlorophyll fluorescence images (**B**), yellowing index and yellowing rate (**C**), L* value (**D**), −a/b ratio (**E**). The data presented in figures indicate as the mean ± standard error of three biological replicates. Asterisks indicate the significant differences (* *p* < 0.05; ** *p* < 0.01) between control and melatonin-treated leaves.

As for pakchoi (fresh produce), maintaining leaves green and freshness is important after harvest, but leaves rapidly yellowing is a major factor affecting its commercial quality. As shown in Figure 1A, the control samples showed yellowing symptoms on the 4th day of shelf life, and then yellowing rapidly. On the 6th day, the commodity value was lost, and on the 8th day, the product turned yellow completely. However, the yellowing process was inhibited by MT treatment, manifesting as slight yellowing was found on the day four, and subsequent changes in the apparent, yellowing rete and yellowing index were relatively slow. The Fv/Fm images (blue area reflected good physiological status) shown in Figure 1B were consistent with the sensory phenotype. The leaf yellowing process was dampened following melatonin application, as evident by the reduced loss of −a/b ratio and inhibited increase of L* value in treated leaves throughout the shelf life (Figure 1D,E).

### 2.2. Exogenous Melatonin Inhibited Chlorophyll Degradation in Pakchoi

**Figure 2:**
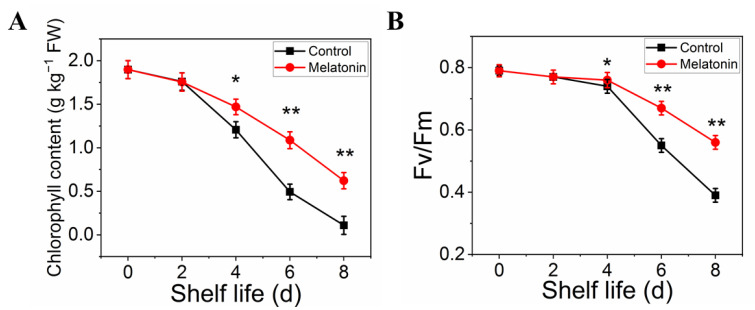
Effects of exogenous melatonin on chlorophyll degradation in postharvest pakchoi. The total chlorophyll content (**A**) and Fv/Fm (**B**). The data presented in figures indicate as the mean ± standard error of three biological replicates. Asterisks indicate the significant differences (* *p* < 0.05; ** *p* < 0.01) between control and melatonin-treated leaves.

The effect of MT treatment on preserving the green color of pakchoi was also confirmed by the results on chlorophyll content. The total chlorophyll content in control leaves declined sharply with the extension of shelf life, it decreased by 95% on the day eight of shelf life. However, the chlorophyll content in treated samples decreased slightly and remained 40% until the end of shelf life (Figure 2A). Moreover, Fv/Fm value (reflect the physiological status in plants) in treated samples remained at a normal level at the end of shelf life, the reduction level was slower than that in control (Figure 2B). 

**Figure 3:**
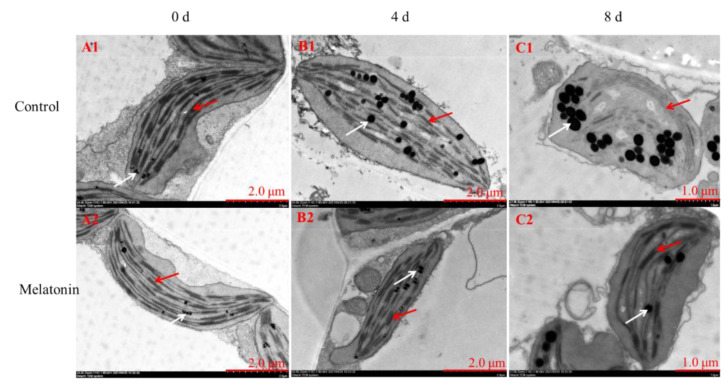
Observation of the chloroplast ultrastructure between control and MT-treated pakchoi leaves on 0 d (**A1**,**A2**), 4 d (**B1**,**B2**) and 8 d of shelf life (**C1**,**C2**). The red arrows indicate the destruction of chloroplast membranes and the white arrows represent the osmiophilic globules, respectively.

Rapid chlorophyll degradation and chloroplast disintegration are generally regarded as biological markers of leaf senescence [4]. In this regard, TEM was used to capture ultrastructural changes. Chloroplasts with a healthy spindle-shaped were observed in newly harvested pakchoi and the lamellar arrangement was clear and regular (Figure 3(A1,A2)). On the 4th day, chloroplasts in control leaves were irregularly shaped, the grana lamellae become sparse, osmiophilic globules become swollen (Figure 3(B1)), and chloroplasts disintegrated severely until the end of shelf life (Figure 3(C1)). However, relative to the control, thylakoid membrane system was maintained well in MT-treated plants during the entire shelf life (Figure 3(B2,C2)). Thus, we found that exogenous application of melatonin inhibited chlorophyll degradation and chloroplast disintegration in postharvest pakchoi.

### 2.3. Exogenous Melatonin Increased Melatonin Accumulation

**Figure 4:**
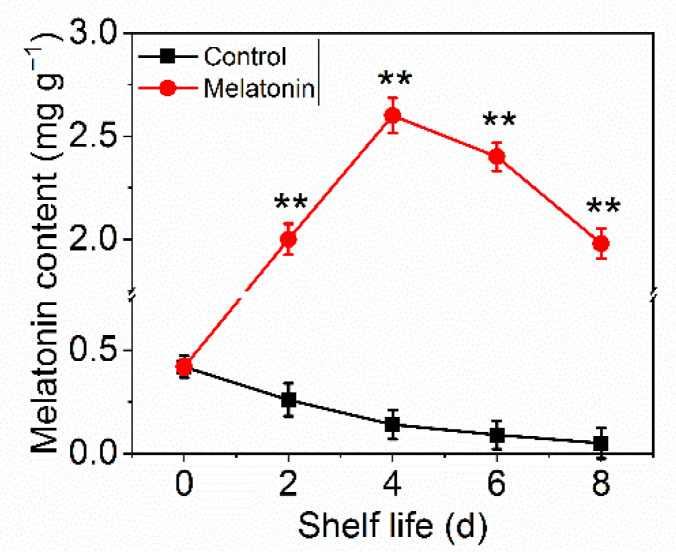
Effects of exogenous melatonin on the melatonin content in control and melatonin-treated leaves. Each value is presented as the mean ± standard error of three biological replicates. Asterisks indicate the significant differences between control and melatonin-treated leaves (** *p* < 0.01).

Melatonin, as a multi-regulatory molecule in plant physiological processes, has been confirmed to be involved in postharvest processes. The content was analyzed to investigate the efficiency of exogenous melatonin effect. From Figure 4, it can be seen that melatonin content in control leaves declined gradually during the shelf life, while that in the treated samples went up greatly and then declined slightly, and was consistently significantly higher than the control group. 

### 2.4. Exogenous Melatonin Mitigated Respiratory Metabolism in Postharvest Pakchoi

**Figure 5:**
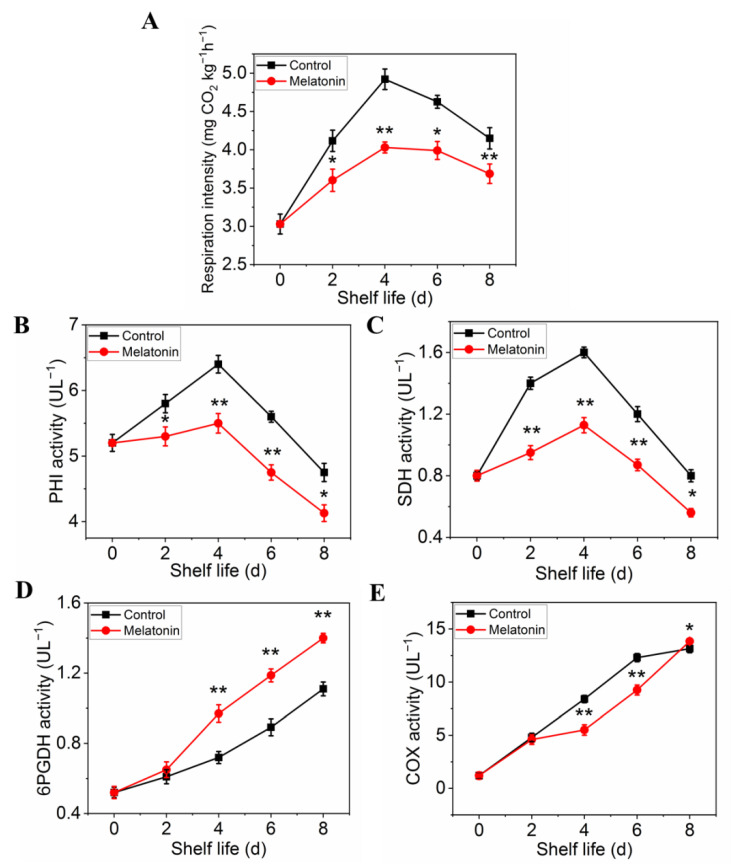
Effects of exogenous melatonin on respiration rate (**A**), respiratory metabolism-related enzyme activities PHI (**B**), SDH (**C**), 6PGDH (**D**) and CCO (**E**). Each value is presented as the mean ± standard error of three biological replicates. Asterisks indicate the significant differences between control and melatonin-treated leaves (* *p* < 0.05 and ** *p* < 0.01).

**Figure 6:**
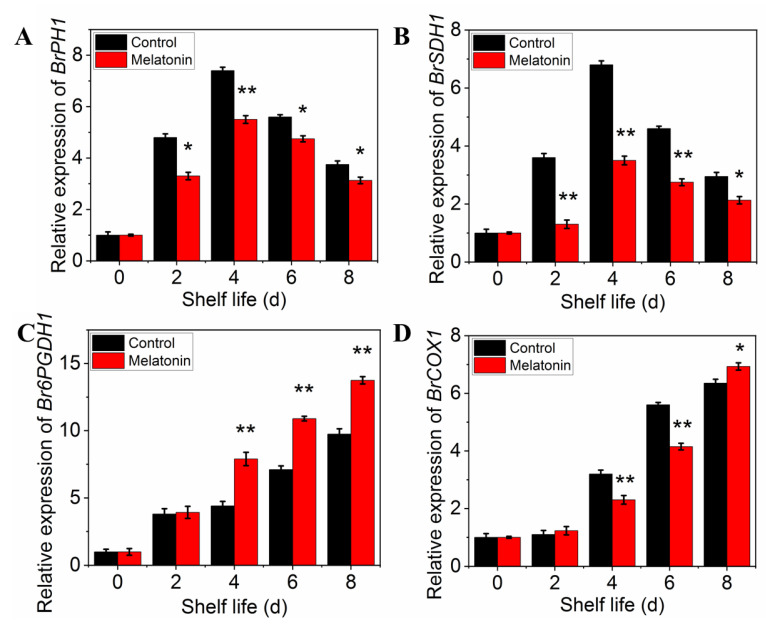
Effects of exogenous melatonin on respiration-related enzyme encoding gene expression in postharvest pakchoi. Changes in the expression level of *BrPHI1* (**A**), *BrSDH1* (**B**), *Br6PGDH1* (**C**) and *BrCOX1* (**D**). Each value is presented as the mean ± standard error of three biological replicates. Asterisks indicate the significant differences between control and melatonin-treated leaves (* *p* < 0.05 and ** *p* < 0.01).

The respiration rate was measured to determine the effect of melatonin on respiratory metabolism. The results presented in Figure 5A showed that the respiration rate of control leaves exhibited a sharp increase at initial and then a slow descent at the end of shelf life. Although the peak of respiratory rate occurred at the same time, the value of the samples treated with melatonin was significantly lower than that of the control throughout the shelf life, indicating that the treatment could effectively inhibit the respiration of the products (Figure 5A). The effect of melatonin on the enzymes activity changes, as well as their gene expression was also investigated. Both PHI and SDH activity in control leaves increased first, reached their peaks on day 4, and then decreased thereafter. The PHI and SDH activity of MT treated samples showed a similar trend, but the range of variation was much smaller than that of the control (Figure 5B,C). The activity of 6PGDH and COX in both control and treated samples increased rapidly with the extension of shelf life, but the activity of 6PGDH in treated samples was higher than that in control (Figure 5D), while the activity of COX was opposite (Figure 5E). The variation in expression levels of ethylene biosynthesis genes was parallel with the enzymes activity (Figure 6A–D).

### 2.5. Exogenous Melatonin Reduced Ethylene Biosynthesis in Postharvest Pakchoi

**Figure 7:**
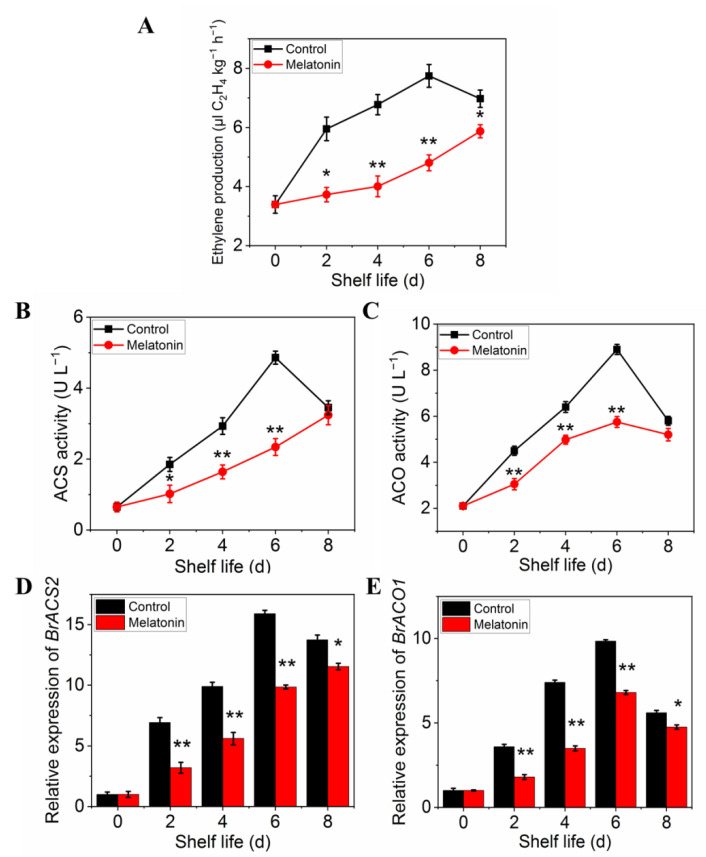
Effects of exogenous melatonin on ethylene biosynthesis in postharvest pakchoi. Changes in ethylene production (**A**), ethylene biosynthesis-related enzyme activities ACS (**B**), ACO (**C**), and the expression of encoding genes, *BrACS2* (**D**) and *BrACO1* (**E**). Each value is presented as the mean ± standard error of three biological replicates. Asterisks indicate the significant differences between control and melatonin-treated leaves (* *p* < 0.05 and ** *p* < 0.01).

The ethylene production of pakchoi was very low at harvest day, but it increased rapidly in control plants from day two of shelf life, reached a maximum at day 6, and then decreased on day 8 (Figure 7A). However, the ethylene production in treated leaves increased slowly, and did not show peak during the entire shelf life. ACS (Figure 7B) and ACO (Figure 7C) activities of untreated pakchoi exhibited similar trajectories with ethylene production: increasing sharply to maxima at 6 d and declining at the final storage time, while it was strongly repressed by MT treatment throughout the entire shelf life (Figure 7C,D). The trends in variation of expression levels were parallel with the enzymes activity (Figure 7D,E). The above results further confirmed that melatonin treatment delayed leaf yellowing in postharvest pakchoi by repressing ethylene biosynthesis.

### 2.6. Correlation Analysis between the Yellowing Index and the Chlorophyll Content, Respiration Rate and Ethylene Production

Based on sensory observing results, we found that the yellowing degree exhibited significant difference between control and treated leaves on the 6th day of shelf life. The correlation analysis was carried out within the first 6 days. The change rate of yellowing index in the control sample was extremely significantly negatively correlated with chlorophyll content, but positively with respiration rate and ethylene production (*r* = −0.992, *p* < 0.01; *r* = 0.962, *p* < 0.01; *r* = 0.793, *p* < 0.01, respectively), indicating that yellowing symptoms directly resulted from chlorophyll loss, and both respiration and ethylene production may accelerate the yellowing process of pakchoi during shelf life. Similar extremely significant correlation was found for MT-treated pakchoi even though the yellowing process, respiration rate and ethylene production were mitigated with MT treatment (Table 1). Therefore, we speculated that the high respiratory activity and more ethylene production may affect the chlorophyll degradation of pakchoi, which in turn led to the deepening of the yellowing process. 

## 3. Discussion

Leaf senescence characterized by leaf yellowing is a major postharvest problem affecting the commercial value and shelf life of leafy vegetables. Pakchoi is particularly prone to leaf yellowing. To delay postharvest yellowing, numerous physical and chemical approaches have been reported and yielded encouraging results, including modified atmosphere packaging [43], 1-MCP application [44], cytokinin dipping [24], NO fumigation [45] and H_2_S dipping [16]. Recently, application of exogenous melatonin has been shown to be a good strategy and a safe method to delay postharvest senescence and reduce postharvest loss [46]. In the present study, the leaf color, chlorophyll content, Fv/Fm and the ultrastructure of the chloroplasts were measured for evaluating the effect of melatonin on leaf yellowing and shelf life of postharvest pakchoi. The results showed that melatonin treatment could effectively delay leaf yellowing and extend the shelf life by inhibiting chlorophyll degradation and chloroplast disintegrated, which was similar to Wu et al. [47] who observed the postharvest yellowing of melatonin treated broccoli was delayed by slowing the chloroplast degradation. Similarly, melatonin treatment delayed postharvest senescence has been reported in Chinese flowering cabbage [6]. The present results may lay the foundation to extend the shelf life and relieve postharvest yellowing of postharvest leafy vegetables using melatonin.

The postharvest loss due to high respiration rate has become the main cause of deterioration in Brassica crops as shown in pakchoi and broccoli [48,49,50]. Prior studies confirmed that higher respiration rate and EMP activity could consume more substrates and accelerate postharvest senescence. In addition, the higher proportion of PPP could dampen senescence in postharvest products by generating more adenosine triphosphate [51]. In this study, the respiration rate, the activities and expression level of PHI, SDH, and COX were apparently inhibited in melatonin-treated leaves, but the higher activities and expression level of 6PGDH were found. This result indicated that melatonin treatment reduced the relative proportion of the EMP pathway and TCA cycle in the total respiratory pathway, but the ratio of PPP was increased in the respiratory pathway, resulting in slowing substrate consumption and delaying the senescence of pakchoi leaves. Tan et al. [6] also found that the reduction of EMP-TCA cycle and enhancement of PPP respiratory pathway might delay the senescence of Chinese flowering cabbage with melatonin treatment. Similar results also found that H_2_S treatment could dampen broccoli senescence by accelerating activity of 6PGDH [51], in agreement with Lin et al. [14], who found that the lower activity of SDH and COX and the higher 6PGDH significantly retarded broccoli postharvest senescence. Meanwhile, our correlation analysis results indicated that yellowing index significantly correlated with respiration rate, suggesting that high respiration rate could consume the nutrient and phytochemical content, accelerated the leaf yellowing process and shortened the shelf life of pakchoi. Thus, melatonin could reduce nutrient consumption, retard leaf yellowing and prolong shelf life in postharvest pakchoi by reduction of the EMP-TCA cycle and enhancement of PPP respiratory pathway. 

Ethylene, as a colorless gas, converted from methionine in plants under the condition of adequate oxygen, can be produced in various parts of plants and distributed in various tissues and organs to promote fruit ripening and leaf senescence. In this study, ethylene production, the activities of ethylene biosynthesis enzyme (ACS and ACO), and their gene expression were significantly suppressed by melatonin treatment. MT reduced ethylene production was found in pear [52] and also in etiolated lupin hypocotyls [53]. A similar result was also found that the crosstalk associated with nitric oxide, H2S and MT can inhibit ethylene production and delay fruit ripening and senescence [54]. In addition, correlation analysis showed a significant positive correlation between ethylene production and yellowing index (*p* < 0.01), but negative for chlorophyll content, indicating that the leaf yellowing resulting from chlorophyll degradation may be closely related to ethylene biosynthesis. It was consistent with a previous study that found that ethylene played a positive role in chlorophyll degradation during leaf senescence [18]. The reduced activity of ethylene biosynthesis enzyme ACS could maintain high chlorophyll content and exhibit a stay-green phenotype in *Arabidopsis* [55]. The mutation of the *EIN2* gene (positive regulator of ethylene signaling) also resulted in more accumulation of chlorophyll content and showed a stay-green phenotype [56]. Treatment with 1-methylcyclopropene (1-MCP, gaseous ethylene antagonist) inhibited broccoli yellowing due to a decrease in Chlase activity [21]. Furthermore, there was an extremely significant positive correlation between respiration rate and ethylene production, suggesting that the respiration of excised vegetable and fruit after harvest could cause autocatalytic ethylene production and accelerate chlorophyll degradation, while the ethylene, could in turn promote the respiration and chlorophyll degradation. The effect of ethylene on respiration rate is a well-known change in most vegetables [57,58] since ethylene can increase the respiration rate irreversibly by regulating respiratory enzyme activity [59,60].The previous study reported that ethylene could cause an increase in respiration rate in broccoli [61], in agreement with Al Ubeed et al. [16], who showed that the addition of ethylene at storage atmosphere increased the respiration rate of postharvest pakchoi during storage. As a consequence of 1-MCP treatment on ethylene production, the respiration rate and the leaf yellowing were also delayed as previously reported in broccoli [1,62,63]. Hence, it is likely that the attenuation of leaf yellowing and prolonging shelf life in MT-treated pakchoi could be associated with the lower respiration rate due to inhibition of ethylene synthesis and action. The more molecule mechanism question of the involvement of ethylene in respiratory metabolism also needs further investigation.

## 4. Materials and Methods

### 4.1. Materials and Treatments

The pakchoi cultivar (cv. ‘huaguan’) with distinct senescence yellowing behaviors was used in this study. Plants at ~45 days of growth were collected from the greenhouse at the Shenyang Agriculture University in October 2021. All plants with uniform color and size (10 cm length), and without mechanical damage, were selected. Pakchoi roots were cut, and the remaining shoots were cleaned with tap water. Harvested samples were transported to laboratory and precooled at 0 °C for 4 h. Based on our previous pilot experiment result, we found the 100 μM of MT was more effective to inhibit leaf yellowing of pakchoi than 50 and 200 μM (Figure S1). Thus, 100 μM was selected in our following treatment experiments. The pakchoi plants were randomly divided into two groups, each of 450 plants and each group within three biological replicates. The plants from MT-treated group were sprayed with 100 μM of melatonin (Sigma-Aldrich, Darmstadt, Germany, CAS#:73-31-4) aqueous solution (containing 0.1% Tween −20). The control group plants were sprayed with distilled water (containing 0.1% Tween −20 (Sigma-Aldrich, Darmstadt, Germany, CAS#:9005-64-5)). Both control and MT-treated plants were air-dried at 25 °C, and then each ten plants packaged in polyethylene bags were placed in a plastic box and were stored at a relative humidity of 80–85% and 25 ± 0.5 °C. Immediately after MT treatment and at 2, 4, 6 and 8 d during the shelf life, 30 plants were taken each time from each group. For consistency, the youngest leaf (center) was defined as leaf 1, and the leaf 6 (second layer medium size leaves) was sampled for the measurement and analysis.

### 4.2. Examination of Leaf Color Changes

The yellowing index evaluation and color parameter quantification were performed as described by Wang et al. [64]. Each five plants from the control and treatment group were selected to determine color parameters at center positions of each 1/4 leaf area in the fresh leaves. The L* value and −a/b ratio were obtained.

### 4.3. Determination of Chlorophyll Content and Fv/Fm 

The leaf tissues of control and treatment group were cut at the same positions as those in Section 2.2. Chlorophyll extraction and concentration calculation were performed as reported previously [3] using a spectrophotometer (DB-20 R, Dynamica, Canberra, Australia). The contents were expressed as g kg ^−1^ FW. For the Fv/Fm, the leaves needed dark-adapt for 15 min and then the images were captured by a FluorCam (Brno, Czech Republic).

### 4.4. Observations of Chloroplast Morphology

Three representative points (0 d, 4 d and 8 d of the shelf life) were selected to transmission electron microscopy analysis. The leaf tissues sampling and fixing were performed as reported previously [64]. The next procedures and images captured were conducted as described in another study [3].

### 4.5. Measurement of Respiration Rate and Ethylene Production

Respiration rate of the control and MT-treated plants was determined based on the CO_2_ accumulation and the O_2_ depletion. Five plants from each replicate per treatment group were sealed in an airtight container (3.2 L) for four hours at room temperature. The concentration of CO_2_ and O_2_ in the container atmosphere was determined using a CheckPoint 3 gas analyzer (PBI Dansensor, Ringsted, Danmark), and results were expressed as mg CO_2_ kg^−1^ h^−1^. Respiration rate was monitored and every two days of shelf life and the measurements were performed in three replicates.

For ethylene production assessment, five plants were weighed and recorded respectively prior to the measurement. Ethylene production was prepared as the same method described as respiration rate. A 1-mL headspace gas sample from each vessel was extracted with a syringe and put into 5-mL sealed glass-jars. The gas samples were monitored every two days of shelf life. Then the samples were injected into a gas chromatograph (CP-3800, Varian, Palo Alto, CA, USA) and the chromatographic conditions were same as the method of Meng et al. [65]. According to the linear regression from ethylene production curves, ethylene production was calculated and expressed in μL kg^−1^ h^−1^.

### 4.6. Assay of Respiratory Metabolism and Ethylene Biosynthesis Related Enzymes Activity

PHI, SDH, 6PGDH and COX were selected as main enzymes involved in plant respiration according to Tan et al. [6] and Guo et al. [66]. The positions of leaf tissues were sampled as mentioned in Section 2.2. The enzyme activities were determined following the method of Tan et al. [6] using the plant assay kits (Keming Biotechnology Co., Ltd., Suzhou, China). The crude enzyme extracting and measuring of ACS and ACO were followed as reported previously [64] using ELISA kits (Enzyme-linked Biotechnology Co., Ltd., Shanghai, China). The results of enzyme activity were expressed as U L^−1^.

### 4.7. Quantitative Real-Time PCR

According to the published results, the respiratory metabolism and ethylene biosynthesis related genes were obtained by Brassica database blast analysis. The full-length CDS sequences were downloaded from Brassicaceae Database (BRAD) and used to design primers. RNA extraction, cDNA synthesis and qRT-PCR were referenced as Wang et al. [67]. Primer sequences in this study were listed in Table S1.

### 4.8. Quantification of Melatonin Content

The extraction and quantification of melatonin were performed as described by Cai et al. [68] by using high-performance liquid chromatography-mass spectrometry (HPLC-MS) (Agilent Technologies, Santa Clara, CA, USA).

### 4.9. Statistical Analysis 

The experiments in this study were performed in triplicate and followed a completely randomized design. Results were expressed as the mean ± standard deviation of three biological replicates. Pearson’s coefficient was used to conduct correlation analysis between different indicators. One-way ANOVA was conducted in SPSS 26.0 (SPSS, Inc., Chicago, IL, USA) to test the significance of treatment effects (2-tailed, *p* < 0.05 or *p* < 0.01). 

## 5. Conclusions

Based on our physiological and biochemical results, the 100 µM MT treatment can markedly retard leaf yellowing in postharvest pakchoi. Specifically, the total chlorophyll, Fv/Fm, and the structural integrity of chloroplast were maintained at a higher level with MT treatment. The respiration, extremely positively correlated with yellowing index, was significantly suppressed in MT-treated leaves, accompanied by decreased activities and expression of PHI, SDH and COX. At the same time, MT treatment effectively inhibited the ethylene production and reduced the activities and expression of ACS and ACO. The correlation analysis verified that leaf yellowing development is closely associated with ethylene production. In summary, the combined effect of respiration and ethylene production can accelerate the yellowing process of pakchoi during shelf life, and MT treatment reduced quality deterioration and alleviated leaf yellowing via reducing respiration consumption and inhibiting ethylene production. In short, our results demonstrated the effectiveness of MT treatment as a preservation method for post-harvested pakchoi to delay leaf yellowing and prolong the shelf life, and thus the use of MT may have broad application prospects for the leafy vegetables. 

## Figures and Tables

**Table 1:** Correlation analysis between the yellowing-index and the chlorophyll content, respiration rate and ethylene production in pakchoi during shelf life.

Correlation Analysis of the Control Pakchoi				
	YellowingIndex	Chlorophyll Content	Respiration Rate	Ethylene Production
**Yellowing index**	1	−0.992 **	0.962 **	0.793 **
**Chlorophyll content**	−0.992 **	1	−0.947 **	−0.788 **
**Respiration rate**	0.962 **	−0.947 **	1	0.835 **
**Ethylene production**	0.793 **	−0.788 **	0.835 **	1
**Correlation analysis of the MT-treated pakchoi**				
**Yellowing index**	1	−0.986 **	0.957 **	0.774 **
**Chlorophyll content**	−0.986 **	1	−0.914 **	−0.754 **
**Respiration rate**	0.957 **	−0.914 **	1	0.787 **
**Ethylene production**	0.774 **	−0.754 **	0.787 **	1

** Correlation is significant at the 0.01 level (two-tailed).

## Data Availability

Not applicable.

## Supplementary Materials

The following supporting information can be downloaded at: https://www.mdpi.com/article/10.3390/plants11162102/s1; Figure S1: Effect of exogenous melatonin with different concentrations on the leaf senescence of pakchoi. Appearance (A), Chlorophyll content (B) and Fv/Fm (C) of control and melatonin-treated leaves during senescence, Table S1: List of primer sequence of qRT-PCR.
