# Supplementary material for: Exogenous Melatonin Alleviated Leaf Yellowing via Inhibiting Respiration and Ethylene Biosynthesis during Shelf Life in Pakchoi
Source: Plants (Basel). 2022 Aug 12;11(16):2102. doi: 10.3390/plants11162102 (PMC9416342; doi:10.3390/plants11162102)
Supplement: Supplementary file 1 [file plants-11-02102-s001.zip › plants-1852160-supplementary.pdf]

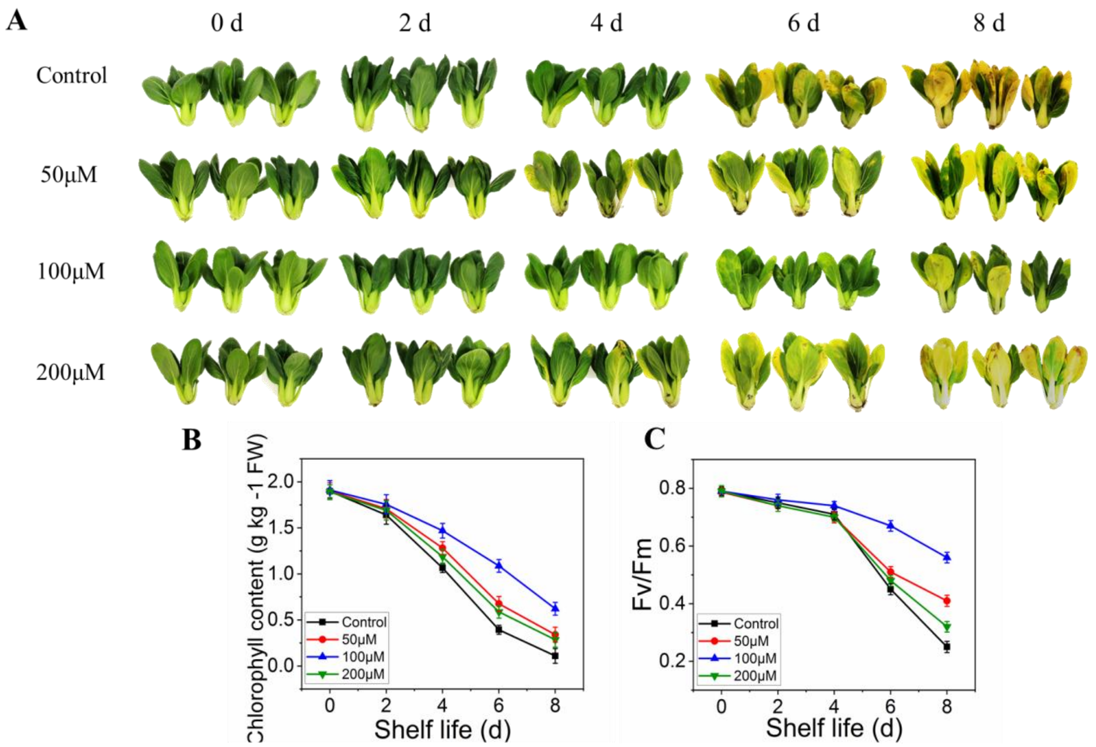

Figure S1 Effect of exogenous melatonin with different concentrations on the leaf senescence of pakchoi. Appearance (A), Chlorophyll content (B) and Fv/Fm (C) of control and melatonin-treated leaves during senescence. Data are the mean ± SE of three biological replicates.

Table S1 List of primer sequence of qRT-PCR.

ID	Primer-Forward	Primer-Reverse
BrACT	CGAAACAACTTACAACTCCA	CTCTTTGCTCATACGGTCA
BrPHI1	AGACGAAAGACACGGGGA	CGGAGGATGAAGGAGGG
BrSDH1	TGGTTCCAGGGCTTATGGCT	CAAGCACGGCCAAACACAAC
Br6PGDH1	CGCGTCGAAAATCTGCAGCT	GCCGACTGCCTCTCGATGAT
BrCOX1	ACATCGAGTTCCACAGGTGC	GAGAGCGCGGTAGTACTTGG
BrACS2	CTCCCGACAAGAATCTGCTG	CGAGGCCCATTTGAATAACA
BrACO1	GGAGATTCCAGTCATTGATTTTAG	TGAGAGTTAATCAGCTGTTTTACTTT
